# Clinical value of intraoperative frozen section examination-assisted strategy in prostate biopsy: a comparative study of diagnostic efficiency and clinical practicality

**DOI:** 10.1186/s12885-026-15960-0

**Published:** 2026-04-13

**Authors:** Yang Luan, Yong-quan Gao, Tian-bao Huang, Liang-yong Zhu, Yong-zhan Gong, Ji Chen, Qin Xiao, Xue-fei Ding

**Affiliations:** Northern Jiangsu People’s Hospital Affiliated to Yangzhou University, No. 98 West Nantong Road, Yangzhou, 225001 Jiangsu Province China

**Keywords:** Prostate cancer, Biopsy, Intraoperative frozen section examination, Number of biopsy cores

## Abstract

**Objective:**

Performing targeted biopsy (TB) first, followed by intraoperative frozen section examination (IFSE), and omitting systematic biopsy (SB) when prostate cancer is detected may reduce the number of unnecessary biopsy cores from SB while maintaining an equivalent prostate cancer detection rate.

**Methods:**

Patients scheduled for mpMRI/TRUS fusion biopsy due to a PIRADS score ≥ 4 were prospectively enrolled between October 2023 and November 2025. Patients were randomly assigned to a control group (TB + SB) or an observation group using a random number table. In the observation group, TB was performed first, and the obtained specimens were immediately subjected to IFSE. If the result was positive, no further cores were taken; if negative, SB was subsequently performed. The positive detection rate, complication rates, and post-radical prostatectomy (RARP) pathological findings were compared between the two groups.

**Results:**

A total of 195 and 187 patients were included in the control and observation groups, respectively. The overall positive biopsy rate was 80.51% (157/195) in the control group and 78.61% (147/187) in the observation group, with no significant statistical difference (*P* = 0.645). The clinically significant prostate cancer (csPCa) detection rates were 67.69% (132/195) and 69.52% (130/187) in the control and observation groups, respectively, showing no significant difference (*P* = 0.701). The mean number of biopsy cores was 23.89 ± 6.42 in the control group and 8.27 ± 8.90 in the observation group, which was significantly lower than the control group (*P* < 0.001). In the observation group, 50 patients (26.74%) underwent immediate additional SB, and 10 (5.35%) had Routine pathology positive for prostate cancer, including 5 with csPCa. The mean time to obtain the final pathology report was 0.025 (0.023,4) days in the observation group, significantly shorter than the 4 (4,5) days in the control group (*P* < 0.001). The VAS pain score was also significantly lower in the observation group compared to the control group (2.52 ± 1.52 vs. 2.91 ± 1.59, *P* = 0.014). Among patients who subsequently underwent RARP, there were no significant differences between the control and observation groups in terms of pathological upgrading/downgrading (44.19% vs. 38.24%) or positive surgical margin rates (25.58% vs. 23.53%) (all *P* > 0.05).

**Conclusion:**

For patients with suspected prostate cancer, the novel biopsy model based on IFSE may be a feasible option. It can maintain a comparable prostate cancer detection rate while significantly reducing the number of biopsy cores, and shows no oncological disadvantage at the time of RARP.

## Introduction

Prostate cancer (PCa) is one of the most prevalent malignant tumors in the male genitourinary system, with an incidence second only to lung cancer in Western countries [1]. In China, its incidence has risen continuously since 2015 and is expected to keep increasing over the next decade, representing a major threat to the health and quality of life of elderly men [2, 3].

At present, prostate biopsy remains the mainstay for the diagnosis of PCa. The combined strategy of multiparametric magnetic resonance imaging (mpMRI)/transrectal ultrasound (TRUS)-guided targeted biopsy (TB) plus systematic biopsy (SB) has been shown to achieve a higher detection rate than either TB or SB alone, especially for clinically significant PCa (csPCa). Accordingly, this combined biopsy approach has been recommended by both domestic and international guidelines [4, 5]. Nevertheless, compared with TB or SB alone, the combined strategy requires a greater number of biopsy cores. Studies have suggested that an increased number of biopsy cores is associated with a higher risk of biopsy-related complications (e.g., urinary tract infection and hematuria) and more severe pain [6–8]. Therefore, optimizing prostate biopsy protocols continues to be an important research focus.

Our research group previously performed intraoperative frozen section examination (IFSE) on TB specimens from patients with a high suspicion of PCa. The results indicated that IFSE could accurately determine the pathological nature of prostate biopsy specimens within a short time [9, 10]. On this basis, the present study aims to perform IFSE on TB specimens. If PCa is diagnosed by IFSE, SB will be omitted; otherwise, SB will be performed subsequently. This modified biopsy strategy is designed to reduce the number of biopsy cores while preserving diagnostic accuracy.

## Materials and methods

### Clinical data

Patients suspected of PCa who were scheduled to undergo mpMRI/TRUS fusion biopsy at Northern Jiangsu People's Hospital Affiliated to Yangzhou University between October 2023 and November 2025 were prospectively recruited. ​

Inclusion criteria: (1) Total prostate-specific antigen (tPSA) ≥ 10 ng/ml; or 4 ng/ml ≤ tPSA < 10 ng/ml with an abnormal free/total PSA ratio (f/tPSA) or abnormal prostate-specific antigen density (PSAD). (2) Prostate Imaging Reporting and Data System (PI-RADS) score ≥ 4. ​

Exclusion criteria: (1) Contraindications to mpMRI examination. (2) Poor or incomplete image quality that precluded accurate analysis. (3) No suspicious lesions identified on mpMRI. (4) A previous history of prostate biopsy or prostate-related treatment (including radiotherapy, chemotherapy, or surgery).

Eligible patients were randomly assigned to the control group or the observation group at a 1:1 ratio using a random number table method. The random sequence was generated by an independent statistician who was not involved in patient enrollment, biopsy procedures, or outcome assessment. Allocation concealment was implemented using sequentially numbered, sealed, and opaque envelopes. Group assignment was revealed only after written informed consent was obtained and eligibility was fully verified, so that neither patients nor treating physicians could predict the allocation in advance, thus minimizing selection bias.

The control group underwent TB + SB, with all collected tissues submitted for routine pathological examination. The observation group first underwent TB, and the obtained tissues were immediately subjected to IFSE. If the IFSE result was positive, SB was omitted; if negative, SB was performed subsequently. No statistically significant differences were observed between the two groups in terms of age, body mass index (BMI), prostate volume, PSA, or PI-RADS score (*P* > 0.05, Table 1). This study was approved by the Ethics Committee of the hospital (Approval No. 2021KY121), and all recruited patients provided written informed consent.

**Table 1:** General data of the patient before prostate biopsy

Characteristics	Observation Group	Control Group	*P -*values
Age (years)	72.30 ± 8.46	71.79 ± 6.60	0.513
BMI (kg/m^2^)	24.33 ± 3.20	24.12 ± 2.79	0.491
Prostate volume (ml)	50.97 ± 28.92	52.14 ± 31.58	0.705
PSA(ng/ml)	31.21 ± 30.88	30.13 ± 29.65	0.908
PI-RADS scores	4(4,5)	4(4,5)	0.194
4	50.80%	57.44%	
5	49.20%	42.56%	

*BMI* Body mass index, *PSA* Prostate-specific antigen, *PI-RADS* Prostate Imaging Report and Data System

### Operational procedure

1. Pre-biopsy preparation: The equipment used included a brachytherapy stepping unit (stabilizer, template, stepper) (Mick Radio-Nuclear Instruments, Mount Vernon, NY, USA), an ultrasound machine (Flex focus 1202; BK Medical, Naerum, Denmark) equipped with a transrectal biplanar probe, and MC1820 disposable biopsy needles (Bard, USA). Oral bowel-cleansing agents were administered preoperatively, and prophylactic antibiotics were not used.
2. Patient positioning and anesthesia: Patients were placed in the lithotomy position. The transrectal ultrasound probe was fixed to the stepper and inserted into the rectum, followed by routine disinfection and draping. Periprostatic nerve block anesthesia was performed using 1% lidocaine [11], and prostate biopsy was initiated 5 min after anesthesia administration.
3. Transperineal prostate biopsy procedure:
  TB: Based on the fusion of mpMRI and TRUS images, 2–3 tissue cores were obtained from each suspicious lesion [9]. The collected tissues were submitted for either IFSE (in the observation group) or routine pathological examination (in the control group).
  SB: The prostate was divided into 11 regions for systematic biopsy [12], with 1–2 tissue cores taken from each region. All tissue samples obtained from SB were sent for routine pathological examination.
4. For some patients with positive biopsy results, robot-assisted radical prostatectomy (RARP) (RARP) [13] was offered as an option. The prostate specimens were examined using whole-mount histological examination.

### Outcome measures

1. Comparison between the two groups included: biopsy positive rate (overall PCa detection rate, csPCa detection rate); average number of biopsy cores required; incidence of complications (hematuria, infection, dysuria); and pain level during biopsy.
2. Based on the results of whole-mount histological examination after RARP, the comparisons between the two groups included: Gleason score changes (upgrading or downgrading) and positive surgical margin rate.
3. Pain level was evaluated using the Visual Analogue Scale (VAS), with the following criteria: 0 points for no pain, 1–3 points for mild pain, 4–6 points for moderate pain, and 7–10 points for severe pain [14]. 

### Statistical analysis

All statistical analyses were performed using SPSS version 2.0 (IBM Corp., Armonk, NY, USA). Continuous variables were tested for normality using the Shapiro- Wilk test. Normally distributed data were presented as mean ± standard deviation (SD) and compared between groups using the independent samples t- test. Non‑normally distributed data were presented as median (interquartile range, IQR) and analyzed using the Mann- Whitney U test. Categorical variables were expressed as frequencies or percentages and compared using the chi- square test or Fisher’s exact test as appropriate. A two- sided *P* < 0.05 was considered statistically significant.

## Results

A total of 382 patients were enrolled in this study, with 195 in the control group and 187 in the observation group.

### Comparison of positive detection rate and csPCa detection rate between the two groups

The overall positive biopsy rate was 80.51% (157/195) in the control group and 78.61% (147/187) in the observation group, with no significant statistical difference between the two groups (*P* = 0.645, Table 2).

**Table 2:** Comparison of prostate biopsy results between the two groups

Groups	Observation Group	Control Group	*P -*values
Overall positive rate (%)	78.61	80.51	0.645
csPCa detection rate (%)	69.52	67.69	0.701
Number of cores	8.27 ± 8.90	24.92 ± 6.16	< 0.001
Time to pathology report (days)	4 (4,5)	0.025 (0.023,4)	< 0.001
Gleason score	8(7,9)	8(7,9)	0.862
6	11.56%	15.92%	
7	33.33%	32.48%	
8	27.89%	26.12%	
9	19.05%	18.47%	
10	8.17%	7.01%	

The csPCa detection rates were 67.69% (132/195) in the control group and 69.52% (130/187) in the observation group, with no significant difference (*P* = 0.701, Table 2).

In the observation group, 50 patients (26.74%) had a negative IFSE result from the TB cores. Among these, 10 patients (5.35% of the observation group) had a positive result on the subsequently performed SB based on routine pathology examination, and 50% (5/10) of these were csPCa. Hence, the sensitivity, specificity, positive predictive value, and negative predictive value of IFSE for diagnosing PCa were 93.2%, 100%, 100%, and 80.0%, respectively, using final conventional pathological results as the reference standard.

### Comparison of biopsy core number, pathological report time and VAS score between the two groups

The mean number of biopsy cores was 23.89 ± 6.42 in the control group and 8.27 ± 8.90 in the observation group, which was significantly lower than that of the control group (*P* < 0.001, Table 2).

The median time to obtain the final pathology report was 4 (4,5) days in the control group and 0.025 (0.023,4) days in the observation group, which was significantly shorter in the observation group (*P* < 0.001, Table 2).

Regarding pain level, the mean VAS score was 2.91 ± 1.59 in the control group and 2.52 ± 1.52 in the observation group, which was significantly lower in the observation group (*P* = 0.014).

### Comparison of Gleason score distribution of biopsy between the two groups

The median GS-B (Gleason score after biopsy) in the control group was 8 (IQR: 7, 9). The distribution of GS-B was as follows: 15.92% (25/157) with score 6, 32.48% (51/157) with score 7, 26.12% (41/157) with score 8, 18.47% (29/157) with score 9, and 7.01% (11/157) with score 10. The median GS-B in the observation group was also 8 (IQR: 7, 9), with a distribution of 11.56% (17/147) score 6, 33.33% (49/147) score 7, 27.89% (41/147) score 8, 19.05% (28/147) score 9, and 8.17% (12/147) score 10. There was no statistically significant difference in the median GS-B between the two groups (*P* = 0.862, Table 2).

### Comparison of the incidence of biopsy complications between the two groups

Regarding biopsy complications, 63 patients (32.31%) in the control group experienced complications, including 54 (27.69%) with hematuria, 4 (2.05%) with infection, and 5 (2.56%) with dysuria. In the observation group, 43 patients (22.99%) had hematuria, 3 (1.60%) had infection, and 4 (2.14%) had dysuria, resulting in an overall complication rate of 26.74%. In the observation group, the 43 patients with complications were distributed in both the IFSE- positive subgroup (who underwent targeted biopsy only) and the IFSE- negative subgroup (who received additional systematic biopsy). Complications occurred during the entire biopsy procedure, including targeted biopsy alone and additional systematic biopsy sampling. There was no significant difference in the overall complication rate compared to the control group (*P* = 0.106).

### Comparison of postoperative pathological results of RARP between the two groups

#### Baseline data of enrolled patients

Subsequently, 86 patients from the control group and 68 patients from the observation group underwent RARP. There were no statistically significant differences between these two subgroups in terms of age, BMI, prostate volume, PSA, PI-RADS score, or biopsy Gleason score (all *P* > 0.05, Table 3).

**Table 3:** General data of the patient before RARP

Characteristics	Observation Group	Control Group	*P -*values
Age (years)	71.34 ± 7.36	70.99 ± 5.69	0.747
BMI (kg/m^2^)	24.57 ± 3.15	24.26 ± 2.84	0.500
Prostate volume (ml)	43.29 ± 18.38	44.60 ± 24.25	0.713
PSA(ng/ml)	23.26 ± 22.40	30.17 ± 23.59	0.067
PI-RADS scores	4(4,5)	5(4,5)	0.169
4	58.82%	47.67%	
5	41.18%	52.33%	
Gleason scores	7(7,8)	7(7,8)	0.877
6	19.12%	17.98%	
7	36.76%	33.72%	
8	20.59%	26.74%	
9	19.12%	15.12	
10	4.41%	5.81%	

#### Postoperative Gleason score distribution of RARP

Whole- mount histological examination results showed that the median GS-R (Gleason scores after RARP) were 7 (IQR: 7, 8) in both the control and observation groups, with no significant difference (*P* = 0.363). In the control group, the GS-R distribution was: 9.30% (8/86) score 6, 50.00% (43/86) score 7, 13.95% (12/86) score 8, 22.09% (19/86) score 9, and 4.65% (4/86) score 10. In the observation group, the GS-R distribution was: 4.41% (3/68) score 6, 45.59% (31/68) score 7, 22.06% (15/68) score 8, 26.47% (18/68) score 9, and 1.47% (1/68) score 10.

#### Pathological upgrading/downgrading after RARP

In this study, pathological upgrading and downgrading were defined by comparing the GS-B with the GS-R (the gold standard for evaluating actual tumor grade): pathological upgrading was referred to as the GS-R being higher than the GS-B, indicating that the actual malignant degree and aggressiveness of the tumor were more severe than the initial evaluation; pathological downgrading was referred to as the GS-R being lower than the GS-B, suggesting that the actual tumor grade was milder than the initial evaluation. Pathological upgrading or downgrading compared to the GS-B occurred in 38 patients (44.19%) in the control group (20 upgraded, 18 downgraded) and in 26 patients (38.24%) in the observation group (19 upgraded, 7 downgraded). There was no significant difference in the rate of Gleason score change between the two groups (*P* = 0.457, Table 4).

**Table 4:** Changes in Gleason scores between the two groups

Gleason scores	6		7		8		9		10		*P -*values
	upgrading	downgrading	upgrading	downgrading	upgrading	downgrading	upgrading	downgrading	upgrading	downgrading	
Control group	10	0	3	2	6	8	1	4	0	4	0.457
Observation group	9	0	5	2	4	3	1	1	0	1	

#### Comparison of positive surgical margin rate after RARP

Positive surgical margins were found in 22 patients (25.58%) in the control group and 16 patients (23.53%) in the observation group, with no significant difference between the groups (*P* = 0.769).

## Discussion

Transperineal template-guided prostate biopsy (TTPB) is one of the most commonly used diagnostic methods for PCa [12, 15]. The traditional prostate biopsy model is SB, which is characterized by simple, flexible operation and real-time monitoring. Although increasing the number of biopsy cores can to a certain extent improve the positive detection rate of SB, some patients at potential risk may still be missed, and some may even require repeated biopsies [16, 17]. With the advancement of mpMRI technology, the diagnostic accuracy for PCa has been significantly improved, with reported sensitivities and specificities as high as 46%- 61% and 79%- 81%, respectively [18, 19]. Consequently, TB has gradually become an important tool for diagnosing PCa, which not only improves the detection rate to a certain extent but also exhibits high diagnostic value particularly for clinically significant prostate cancer (csPCa) [20–22]. However, a negative mpMRI result cannot completely rule out the possibility of PCa. Additionally, due to the multifocal nature of PCa, TB may still miss 16.0%- 25.4% of csPCa cases [23, 24]. The results of our study also showed that 26.74% (50/187) of patients in the observation group underwent additional SB based on a negative IFSE result. Among these patients, 20% (10/50) were diagnosed with PCa after the additional SB, and 50% (5/10) of these cases were csPCa. Therefore, both international and domestic guidelines, as well as most relevant studies, recommend the combined TB + SB approach to further improve the detection rate of PCa [4, 5, 25].

Nevertheless, combining SB with TB inevitably increases the number of biopsy cores. Studies have demonstrated that the risk of severe hematuria and infection associated with TTPB is correlated with an increased number of biopsy cores [6, 26]. Furthermore, an increased number of biopsy cores leads to additive pain and higher pain scores [7]. This necessitates robust anesthesia techniques prior to biopsy, such as periprostatic nerve block, spinal anesthesia, or even general anesthesia, which imposes higher requirements on the patient’s physical condition. How to reduce the number of biopsy cores as much as possible without compromising the PCa detection rate remains a relentless pursuit for urologists.

Compared to traditional paraffin embedding and sectioning, IFSE eliminates the need for fixation, dehydration, and embedding, allowing for direct sectioning, saving time, and being both rapid and simple. During the rapid freezing process, it can well-preserve the activity of various antigens and enzymes, especially those sensitive to temperature and organic solvents, reducing structural alterations in the tissue and improving diagnostic accuracy. This technique has been widely applied in breast, lung, uterine, and thyroid tissues [27–29]. Our research group previously applied IFSE to prostate biopsy, and the results showed that IFSE could ensure diagnostic accuracy to a certain extent while rapidly providing pathological results [9, 10].

Therefore, to avoid missed diagnoses associated with TB alone and reduce the complications and pain caused by the increased number of biopsy cores in the TB + SB approach, this study adopted a modified strategy: TB was performed first, followed by IFSE on the obtained tissues. When the IFSE result was not definitively positive (including benign findings or uncertain diagnoses), additional SB was performed, with all tissues submitted for routine pathological examination. The primary objective of this modified approach was to reduce the number of biopsy cores while preventing missed diagnoses.

The results of this study demonstrated that the overall biopsy positive rates were 80.51% (157/195) in the control group and 78.61% (147/187) in the observation group, with csPCa detection rates of 67.69% (132/195) and 69.52% (130/187), respectively. No statistically significant differences were observed in these detection rates between the two groups (P > 0.05), nor in the Gleason scores of patients with positive biopsy results (*P* > 0.05). However, the mean number of biopsy cores was 23.89 ± 6.42 in the control group versus 8.27 ± 8.90 in the observation group, a difference that was statistically significant (*P* < 0.001). More importantly, the mean VAS score was 2.91 ± 1.59 in the control group and 2.52 ± 1.52 in the observation group, with the latter being significantly lower (*P* = 0.014). Thus, the biopsy strategy used in the observation group significantly reduced both the number of biopsy cores and the procedural pain experienced by patients. Furthermore, the turnaround time for obtaining the final pathology report was significantly shorter in the observation group compared with the control group (0.025 (0.023,4) days vs. 4 (4, 5)days, *P* < 0.001). This may facilitate earlier clinical decision- making and help streamline clinical pathways, which could indirectly contribute to more efficient hospital management. In addition, the reduced waiting period may reduce patient uncertainty and psychological distress associated with diagnostic waiting, although formal assessment of patient‑reported outcomes was not performed in this study.

Ultimately, 154 patients underwent RARP, including 86 in the control group and 68 in the observation group. No statistically significant differences were noted in the preoperative baseline characteristics between the two groups (*P* > 0.05). Compared with the results of whole-mount histological examination after RARP, pathological upgrading or downgrading occurred in 38 patients (44.19%) in the control group and 26 patients (38.24%) in the observation group, with no statistically significant difference between the two groups (*P* = 0.457). A further comparison of positive surgical margin rates revealed 25.58% (22/86) in the control group and 23.53% (16/68) in the observation group, which also showed no statistically significant difference (*P* = 0.769).

This study has several limitations that should be acknowledged. First, this novel biopsy strategy modifies the conventional surgical workflow. Waiting for IFSE results prolongs the total procedure time and moderately reduces operating room turnover efficiency. Second, the additional expenses for IFSE consumables, pathological testing, and dedicated pathologist labor may moderately increase procedure-related costs for patients with negative IFSE who subsequently undergo systematic biopsy. Third, this IFSE-based protocol requires a well-established intraoperative pathology support system and is therefore more suitable for implementation in high-volume medical centers with dedicated genitourinary pathologists and timely IFSE services. Fourth, length of hospital stay, hospitalization costs, and patients’ psychological burden were not systematically evaluated; therefore, its comprehensive health-economic and patient-centered benefits warrant further validation.

In summary, for patients with mpMRI findings suggestive of PCa, the Optimized prostate biopsy protocol assisted by IFSE can reduce the number of biopsy cores while achieving a detection rate comparable to that of SB + TB. Furthermore, this Optimized strategy demonstrated no relevant oncological disadvantages in patients who underwent RARP. Collectively, this IFSE-assisted biopsy approach is safe, feasible, and holds significant potential for clinical application and promotion.

## Data Availability

The datasets generated and analyzed during the current study are available from the corresponding author upon reasonable request.
